# Bioconversion of cow manure through vermicomposting: effects of tylosin concentration on the weight of worms and manure quality

**DOI:** 10.1038/s41598-024-62839-w

**Published:** 2024-05-31

**Authors:** Farnaz Ghandehari Yazdi, Mehdi Mokhtari, Mohsen Nabi Meibodi, Reyhane Sefidkar, Behnam Hatami, Fereshteh Molavi, Mahin Ghafourzadeh, Ahmad Golshiri, Ali Asghar Ebrahimi

**Affiliations:** 1 Environmental Science and Technology Research Center, Department of Environmental Health Engineering, School of Public Health, Shahid Sadoughi University of Medical Sciences, Yazd, Iran; 2 Department of Pharmaceutics, Faculty of Pharmacy, Shahid Sadoughi University of Medical Sciences, Yazd, Iran; 3 Center for Healthcare Data Modeling, Department of Biostatistics and Epidemiology, School of Public Health, Shahid Sadoughi University of Medical Sciences, Yazd, Iran; 4 Department of Medical Parasitology & Mycology, Paramedical School, Shahid Sadoughi University of Medical Sciences, Yazd, Iran

**Keywords:** Vermicomposting, Animal manure, Tylosin antibiotic, Earthworm weight, Bioconversion, Environmental sciences, Environmental social sciences

## Abstract

This study investigated batch-fed vermicomposting of cow manure, with a specific focus on assessing the effects of tylosin on the weight of earthworms and the overall quality of the resulting manure. Five reactors, including three concentrations of tylosin (50, 100, and 150 mg/kg) and two control reactors, were employed. Residual tylosin concentrations were measured using high-performance liquid chromatography (HPLC). Quality parameters such as pH, temperature, volatile solids (VS), organic carbon content (OCC), electrical conductivity (EC), ash content, C/N ratio, total Kjeldahl nitrogen (TKN), and microbial content were evaluated. The toxicity and maturity of vermicompost were assessed by determining the germination index (GI). The study also monitored variations in the earthworm’s weight. The results demonstrated a decreasing trend in VS, OCC, C/N, and fecal coliforms, along with increased pH, EC, ash content, and TKN during the vermicomposting process. Furthermore, investigations revealed significant reductions in the reactors with tylosin concentrations of 50, 100, and 150 mg/kg, resulting in the removal of 98%, 90.48%, and 89.38% of the initial tylosin, respectively. This result confirms the faster removal of tylosin in reactors with lower concentrations. Degradation of tylosin also conforms to first-order kinetics. The findings showed a significant influence of tylosin on the weight of *Eisenia fetida* earthworms and the lowest antibiotic concentration led to the highest weight gain. Finally, the high percentage of germination index (90–100%) showed that the quality and maturity of vermicompost is by national and international standards.

## Introduction

Since the inception of antibiotic exploration in the early 1900s, these agents have been employed for a myriad of purposes, including the treatment of infectious diseases and growth promotion in domesticated animals^1^. Upon administration, a fractional portion of antibiotics is assimilated, with 30–90% subsequently excreted through fecal and urinary pathways as parent compounds or bioactive metabolites. Non-metabolized antibiotics persist in animal manure, leading to the emergence of antibiotic-resistant microorganisms in the surrounding environment^2,3^. The indiscriminate application of antibiotics engenders manifold risks for human society, including the proliferation of antibiotic-resistant microorganisms, the generation of novel serotypes exhibiting diverse pathogenicity, the induction of allergic reactions in susceptible individuals, disruption of fermentation processes in the production of dairy products, and complications in diagnostic procedures within medical and food laboratories^4^.

In recent years, the pervasive use of antibiotics in veterinary practices has precipitated global apprehension regarding antibiotics and their associated resistance genes^3^. Consequently, the imperative for the eradication of antibiotics has become exceptionally pronounced. The chemical properties of antibiotics and the characteristics inherent in manure determine their resilience to biodegradation and elimination. Diverse categories of antibiotics, including beta-lactam antibiotics, macrolides, sulfonamides, trimethoprim, fluoroquinolones, tetracyclines, and polyether antibiotics^5^, have been applied across various livestock industries. In particular, macrolides, which are known for their antimicrobial efficacy against Gram-positive bacteria, are particularly important. Macrolides are delineated based on the number of lactone ring atoms, ranging from 12 to 16 atoms^6^.

Tylosin, a pivotal macrolide, commands widespread application in veterinary medicine globally, leading to the annual generation of millions of tons of fermentation residue waste^7^. Tylosin, characterized by a 16-membered ring macrolide structure, consists of four distinct substances: tylosin A, tylosin B (desmycosin), tylosin C (macrocin), and tylosin D (relomycin)^6^.

In the proposed guidelines outlined in the US Food and Drug Administration’s Food Safety Modernization Act (FSMA), adherence to a manure treatment process capable of diminishing the prevalence of identified pathogens before land application is deemed acceptable^8^. Until now, various methods such as gamma rays, microwaves, heat, ultraviolet rays, oxidation, and composting have been employed for antibiotic removal^9^. Notably, vermicomposting has emerged as a conspicuous contender among these methods. In contrast to traditional composting, vermicomposting has a higher concentration of soluble nutrients and a higher organic matter content. This method involves a bio-oxidative mechanism in which earthworms, microorganisms, and other decomposer communities synergistically engage, expediting organic waste degradation^10^. In the vermicomposting paradigm, earthworms digest and metamorphose organic waste simultaneously, yielding compost suitable for crop cultivation. Notably, earthworm metabolic activities increase the nutrient content of the converted wastes. Finally, vermicomposting yields a surplus of available nutrients per unit weight compared with the initial organic substrate used by the earthworms.

In a meticulous examination of the confluence between tylosin, a notable veterinary antibiotic, and vermicomposting, we explore the intricate dynamics governing sustainable waste management methodologies. This inquiry entails a thorough assessment of Tylosin's impact, spanning from its effects on earthworms to the chemical and microbial characteristics of vermicompost manure. The primary objective of this focused investigation is not only to enhance the comprehension of eco-friendly waste management but also to delineate actionable insights for alleviating antibiotic residues in animal manure. This, in turn, contributes to the advancement of environmentally conscious agricultural practices.

## Materials and methods

### Raw manure and chemicals

The initial raw material for vermicomposting was obtained from the manure storage tank of a livestock facility in Yazd City, Iran. Before starting the experiment, the raw materials underwent meticulous mixing, which was conducted 2–3 times to achieve a reasonably homogeneous composition. The earthworm *Eisenia fetida* was procured from the Agricultural Jahad Organization of Yazd Province, Iran. Notably, these earthworms were in an active state upon introduction to the substrate. The tylosin used in the study, characterized by 99% purity and bearing the CAS number 1401-69-0, was sourced from Royandarou Co., located in Tehran, Iran.

### Earthworm acclimatization

This study encompassed two key phases: an initial stage dedicated to the acclimatization of earthworms, followed by the primary vermicomposting process. The initial 2-week period focused on the careful adjustment of earthworms to a fertilizer-enriched environment. This was succeeded by an additional 4-week phase in a separate pilot study, during which the earthworms were exposed to antibiotics. Following the acclimatization phases, the primary vermicomposting process commenced, serving as the focal point and spanning 60 days.

### Vermicomposting setup

**Figure 1:**
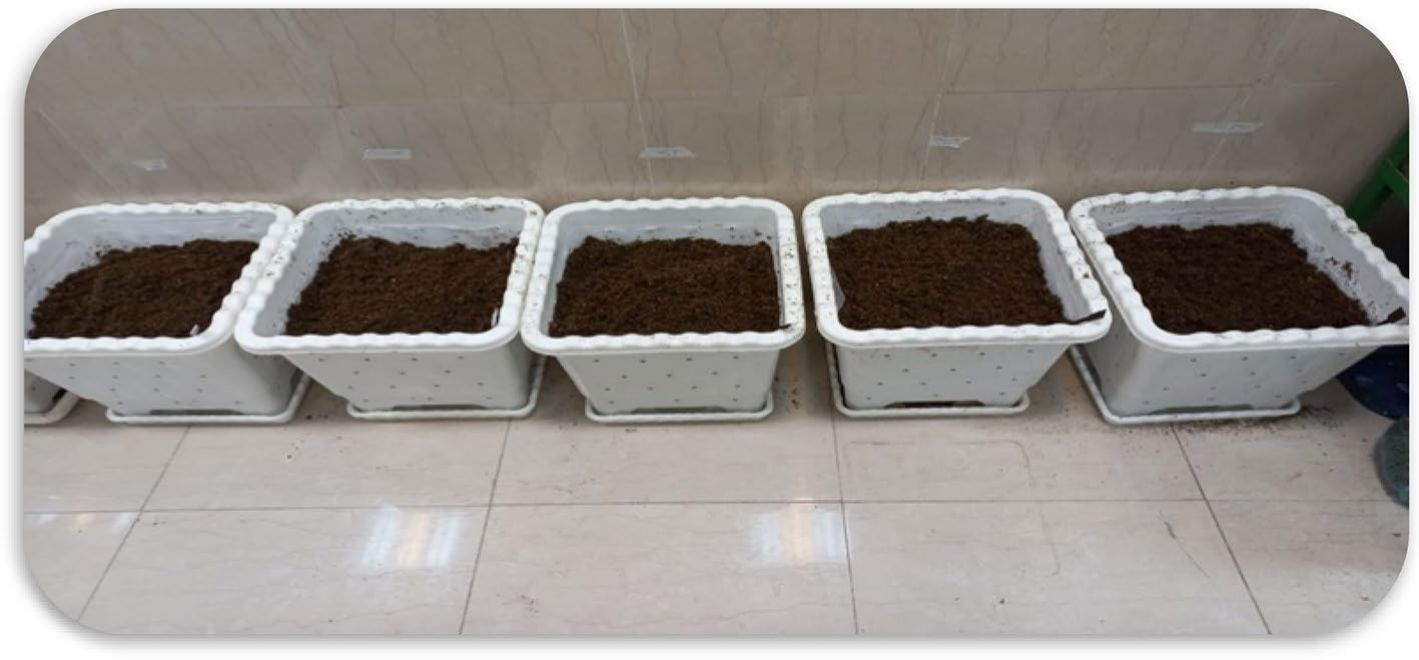
The pilot-scale vermicomposting system used in this study.

The vermicomposting process was conducted in five plastic containers (30 × 30 × 30 cm) (Fig. 1). To facilitate an oxygen-rich environment, perforations were strategically implemented at the base and floors of each reactor. The earthworm substrate was cow manure, which underwent thorough washing with distilled water to mitigate the presence of ammonia and urea, which are potential threats to earthworm vitality. Each reactor received 2.33 kg of cow manure, and 120 g of earthworms were introduced into each reactor, totaling approximately 280–300 earthworms per reactor. Throughout the process, the ambient temperature and humidity were consistently maintained at 23–25 °C and approximately 60%, respectively, across all reactors.

Tylosin was incorporated into the reactors at 50, 100, and 150 mg/kg concentrations. The antibiotic, dissolved in distilled water, was uniformly sprayed onto the manure. Two control reactors were included: one without antibiotics but with earthworms and another devoid of earthworms but containing a 100 mg/kg antibiotic concentration. Sampling adhered to a predetermined schedule, and after 63 days, earthworms were harvested from the vermicompost.

### Physicochemical and microbial properties and antibiotic analysis

In this study, various physicochemical properties, including volatile solids (VS), organic carbon content, ash content, electrical conductivity (EC), pH, temperature, and total Kjeldahl nitrogen (TKN), were assessed to determine the effectiveness of the vermicomposting process. Moreover, the microbial properties of the samples, including fecal coliforms and parasite eggs, were measured. Analyses mentioned above were conducted according to the National ICS guidelines (Iranian National Standards Organization)^11^.

The extraction of antibiotics followed the solid-phase extraction methodology described by Yue et al.^12^. Tylosin residues were determined employing high-performance liquid chromatography equipped with a UV/Vis detector and a C18 column at 254 nm. The mobile phase in this study was water–acetonitrile (0.04 M) and phosphate buffer (pH = 6) at a 34:66 ratio. In this study, concentrations of 25, 50, 75, 100, 150, and 300 μg/ml were used to prepare the standard curve of the tylosin antibiotic. The degradation kinetics of tylosin at various initial concentrations were modeled using the following first-order kinetic expression:

1 $$C={C}_{0}{e}^{-kt},$$

where $k$ represents the rate constant for tylosin degradation (h–1); $C$ and ${C}_{0}$ are the tylosin concentration (mg/L) at time $t$ (h) and the initial tylosin concentration (mg/L), respectively. Finally, the efficiency of tylosin removal was computed as follows:

2 $$Removal \,\,efficiency \left(\%\right)=\frac{{C}_{0}-C}{{C}_{0}}\times 100 .$$

### Toxicity and maturity assessment of vermicompost's manure

Germination index is one of the most essential sensitive biological parameters for evaluating the toxicity and maturity of vermicompost. It is determined by the ability of vegetables to germinate under suitable environmental conditions. To this end, a volume of 50 cm^3^ from the sample was transferred to a 150-ml beaker, and distilled water was subsequently added to double its volume. The mixture was thoroughly mixed and stood for 2–3 h. This mixture was filtered. Next, the filtered extract (10 ml) was added to a Petri dish containing 10 Garden Cress seeds and placed in a dark environment for 24–48 h. A control sample was prepared using distilled water following the same procedure. Finally, the count of germinated seeds in both the sample and control groups was compared^13^.

### Statistical analyses

In this research, IBM SPSS Statistics, version 22.0 software (IBM Corp., Armonk, N.Y., USA, https://www.ibm.com/spss) was used for data analysis, and Origin 2019 software was used to draw graphs. One-way analysis of variance (ANOVA) was used considering the significance level of 95% (p-value ≤ 0.05).

## Results and discussion

### Changes in physicochemical and microbial properties during vermicomposting

#### pH

**Figure 2:**
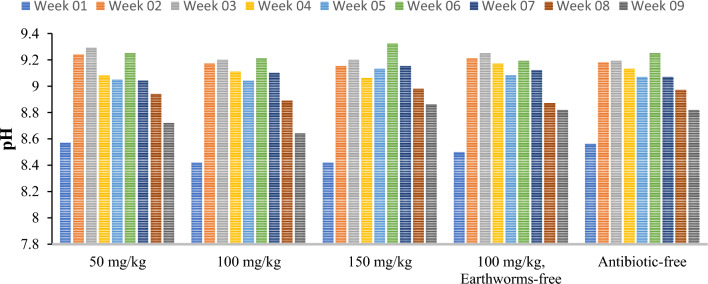
Observed variations in pH throughout the vermicomposting duration in the studied reactors (50, 100, 150 mg/kg tylosin, 100 mg/kg tylosin without earthworm, and antibiotic-free reactor).

The pH of manure is another important environmental factor that affects the vermicomposting process. In this study, reactor type did not significantly affect the average pH value (p = 0.32). However, vermicomposting duration exhibited a significant impact on pH changes (p < 0.001), with an observed increasing trend from the first to ninth week (Fig. 2). This can be attributed to the consumption of hydrogen ions by various hydroxyl groups and phenols during the mineralization and purification of soil organic carbon, along with a decrease in volatile acids during vermicomposting^14,15^. pH reduction can also be related to nitrogen and phosphorus mineralization and microbial decomposition activities^16^. In this regard, our study’s results align with Molavi et al. and A’Ali et al.^17,18^.

#### Volatile solids (VS) and ash content

**Figure 3:**
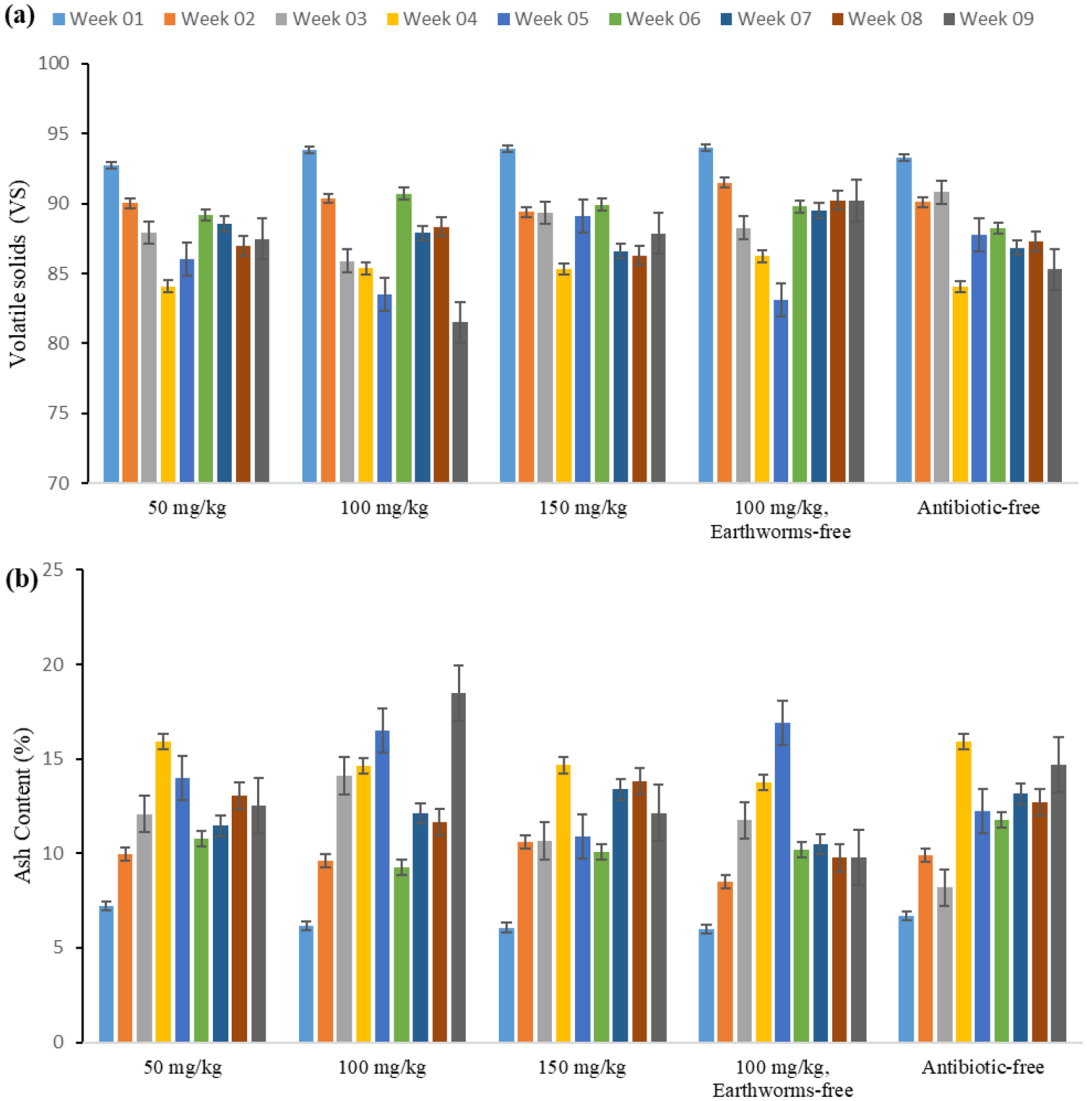
Observed variations in (**a**) volatile solids, and (**b**) ash content throughout the vermicomposting duration in the studied reactors (50, 100, 150 mg/kg tylosin, 100 mg/kg tylosin without earthworm, and antibiotic-free reactor).

The decrease in volatile solids is a crucial indicator for assessing the treatment efficacy of vermicompost. It indeed verifies the enhancement of decomposition and mineralization in the presence of earthworms^19^. The improvement of the microclimate due to the activity of earthworms leads to the creation of a suitable environment for increasing the activity of microorganisms, which also increases the biological decomposition of organic materials leading to a further reduction in volatile solids^20,21^. The trend of changes in the total solids is presented in Fig. 3. As can be seen, the reactor type did not have a significant effect on the average VS value (p = 0.308). Instead, vermicomposting duration exhibited a substantial impact on VS changes (p < 0.001). The volatile solids at the beginning of the process in all studied reactors were approximately 92–94%. These values decreased until the fifth week, experienced a subsequent increase, and then until the end of the process, it had a decreasing trend of 5–14%. These findings confirm the pivotal role of earthworms in vermicomposting^21^ and are consistent with the results of Molavi et al. in 2020 and Ujwala Hujuri et al. in 2022^17,22^. Both studies reported a decreasing trend in volatile solids at the end of the process.

Ash content is an important indicator for evaluating the decomposition and mineralization of materials during the vermicomposting process^23^. This index confirms the degradation of glucose, hemicellulose, and lignin during decomposition, accompanied by the simultaneous release of carbon dioxide^24^. Furthermore, the rise in ash content can be related to increasing microbial activity in the logarithmic phase and earthworm activity due to the presence of a favorable soft substrate^25^. The trend of changes in the ash content is depicted in Fig. 3. As can be seen, the type of reactor did not significantly affect the average ash content value (p = 0.347). Conversely, the duration of vermicomposting had a significant impact on changes in the ash content (p < 0.001). At the beginning of the process, the ash content was approximately 5–7%, and by the end of the process, it had increased to 18–19%, showing an overall upward trend in changes. This observed increase in the ash content indicates that worms consumed organic materials at an accelerated rate and that the microbial community effectively carried out decomposition activities^26^.

#### Organic carbon, and electrical conductivity (EC)

**Figure 4:**
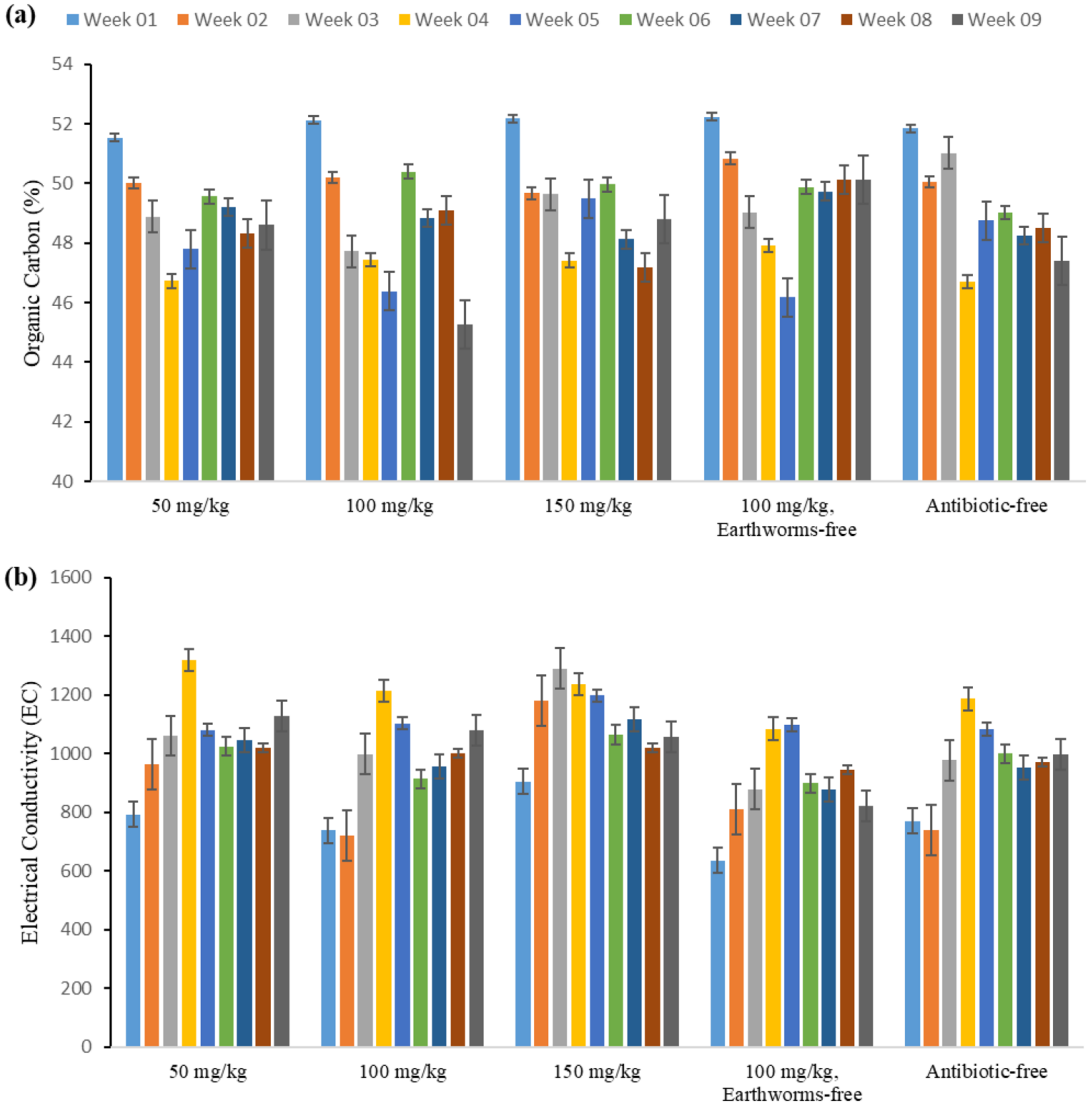
Observed variations in (**a**) organic carbon, and (**b**) electrical conductivity throughout the vermicomposting duration in the studied reactors (50, 100, 150 mg/kg tylosin, 100 mg/kg tylosin without earthworm, and antibiotic-free reactor).

According to the statistical analysis results, the type of reactor did not significantly affect the organic carbon content (p = 0.396). In contrast, the duration of vermicomposting had a significant impact on changes in organic carbon content (p < 0.001). Figure 4 illustrates the changes in the amount of organic carbon in the studied reactors. At the start of the process, the organic carbon content in the reactors was approximately 52–51%. After four to five weeks, it decreased to approximately 46–47%, and at the end of the process, it reached approximately 45–50%. One of the reasons for the reduction in organic carbon is that it is released in the form of carbon dioxide during microbial respiration and the mineralization of organic materials as a result of the increase in total nitrogen. Part of the carbon in the decomposed remains is released as carbon dioxide, while another part is absorbed by the microbial biomass^27^. A study by Sierra et al. observed a decreasing trend in the percentage of organic carbon during their investigation of composting and vermicomposting of cow manure and green waste, findings that align with our current study^28^.

The number of cations and anions in the compost mass was determined by measuring its electrical conductivity. Electrical conductivity is an indicator of the salinity of organic compounds. Elevated salt concentrations may lead to plant toxicity issues. Therefore, electrical conductivity is a crucial parameter indicating the suitability and safety of compost or vermicompost^29^. In this study, the electrical conductivity in all reactors increased, as shown in Fig. 4. The initial values ranged from 580 to 730 μS/cm and increased during the process, reaching over 1000 μS/cm at the end of the process in reactors containing earthworms. According to the statistical analysis results, both the type of reactor and the duration of vermicomposting had a significant impact on changes in EC (p < 0.001). Using the ninth week as the reference time, the statistical results indicated a significant difference in the average electrical conductivity in the first week (p < 0.001), second week (p = 0.002), and fourth week (p < 0.013) compared with the ninth week. Additionally, the results of follow-up tests revealed a significant disparity in average electrical conductivity among reactors treated with different concentrations of tylosin. Specifically, in comparison to the control group with earthworms but without antibiotics, reactors containing 50 mg/kg (p = 0.047), 100 mg/kg (p = 0.014), and 150 mg/kg (p = 0.001) of tylosin exhibited notable variations in electrical conductivity. Furthermore, upon further comparison, a significant difference was observed in the average electrical conductivity between reactors treated with 150 mg/kg of tylosin and those treated with 100 mg/kg (p < 0.001) and 50 mg/kg (p > 0.001) of tylosin. Similarly, a significant difference was noted between reactors treated with 100 mg/kg of tylosin and those treated with 50 mg/kg of tylosin (p = 0.004), all in comparison to the control group with 100 mg/kg of tylosin but without earthworms.

The increase in electrical conductivity can be linked to mineralization and the rise in soluble salts^30,31^. Another contributing factor to the increase is the breakdown of organic materials and the release of mineral elements such as calcium, magnesium, potassium, and phosphorus in their exchangeable forms, i.e. as cations in vermicompost and compost^27^. Additionally, the increase in electrical conductivity may result from the loss of weight of organic materials and the release of various mineral salts in their existing forms, such as phosphate, ammonium, and potassium, as reported by other researchers^32^. The findings of our study align with those of Meena Khwairakpam et al. and Ramalingam Balachandar et al.^30,32^.

#### Carbon-to-nitrogen (C/N) ratio, and total Kjeldahl nitrogen (TKN)

**Figure 5:**
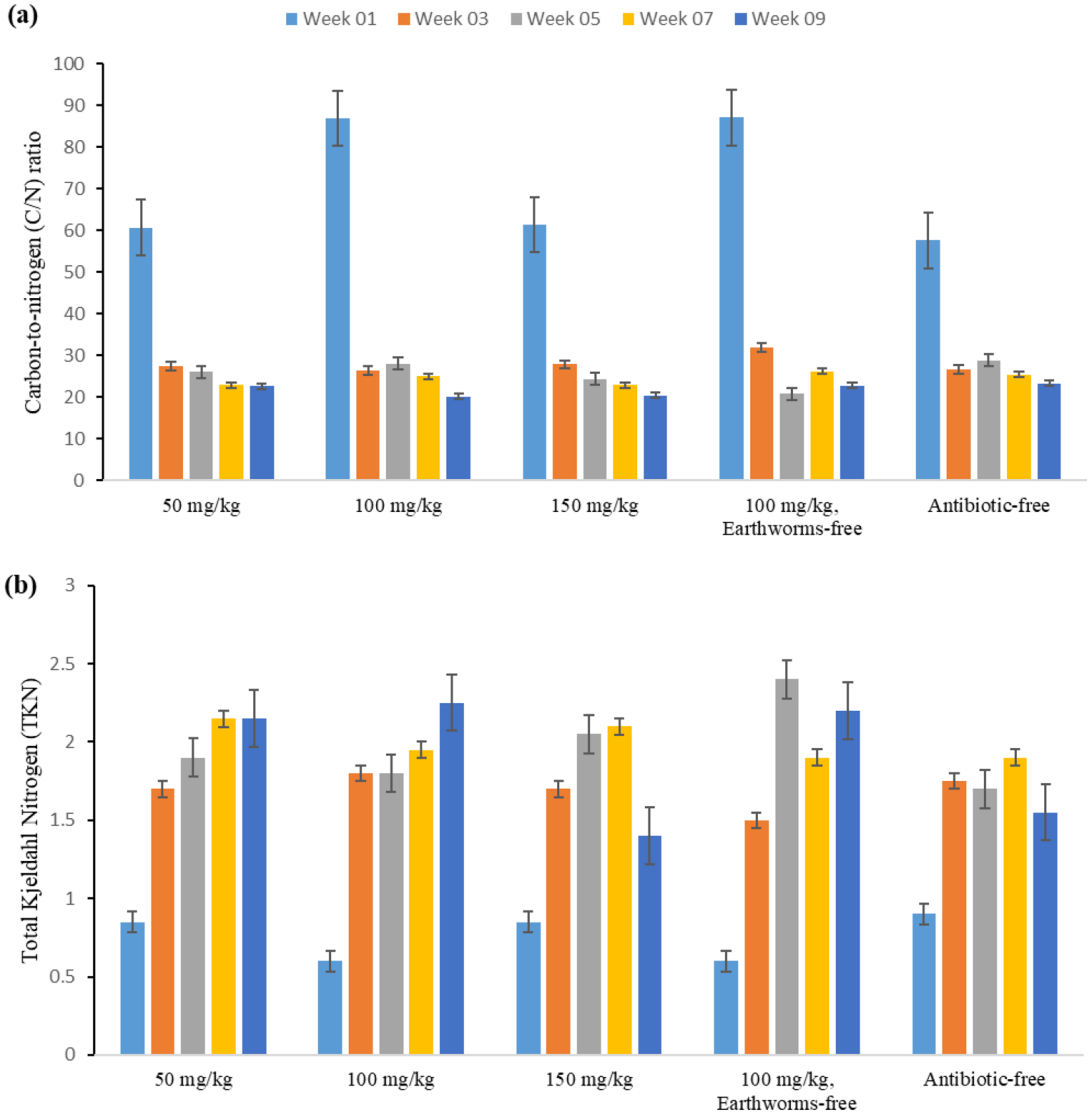
Observed variations in (**a**) Carbon-to-nitrogen (C/N) ratio, and (**b**) Total Kjeldahl Nitrogen (TKN) throughout the vermicomposting duration in the studied reactors (50, 100, 150 mg/kg tylosin, 100 mg/kg tylosin without earthworm, and antibiotic-free reactor).

The carbon-to-nitrogen ratio plays a crucial role in improving physical, chemical, and biological activities, in addition to supporting crop growth. Typically, the carbon-to-nitrogen ratio tends to decrease because of the decomposition of organic matter and the reduction of organic carbon. A lower C/N ratio accelerates the decomposition of organic matter, consequently increasing the nitrogen content^33^. The reduction in organic carbon during the vermicomposting process is primarily associated with the respiratory activities of microorganisms and worms, concomitant with an increase in nitrogen from mucus and excretory sources^34^. In this study, as illustrated in Fig. 5, the observed changes exhibit a decreasing trend and reach their standard range by the end of the 9 weeks. The ideal carbon–nitrogen ratio standard for first-grade compost is approximately 15–25, which was observed in our study. The findings of our study agree with those of previous research conducted by Wang et al.^35^.

Nitrogen, an essential nutrient for plant growth, is important in the final assessment of manure quality. According to the statistical analysis results, the duration of vermicomposting had a significant impact on the changes in TKN (p < 0.001). Figure 5 illustrates an increase in the final TKN values compared to the initial values across all reactors, aligning with the findings of Ashok Kumar et al. and Yatoo et al.^36,37^. Factors affecting the increase of TCN levels during decomposition by microorganisms include the reduction of organic matter, the conversion of carbon from organic matter to CO_2_, and the decrease of humidity due to evaporation during mineralization^33^. Furthermore, the nitrogen content in earthworm mucus, body fluids, enzymes, and the decayed tissues of earthworms, as well as the carbon-rich mucus and excrement products of earthworms, can create an environment conducive to increased nitrogen content in the soil^16^. The results from the conducted studies reveal that the nitrification rate may be enhanced during the maturation stage of vermicompost due to low concentrations of antibiotics, but high concentrations of antibiotics can inhibit this process. In this study, at a dosage of 50 mg/kg tylosin antibiotics, we detected slightly higher levels of nitrogen content than at 150 mg/kg. This stability in results could be an indication of a potential trend. Nevertheless, the literature has demonstrated variation in the results of other studies, most probably because of the type and amount of antibiotics used^38^.

#### Fecal coliforms and parasite eggs

The reduction and destruction of pathogens during vermicomposting can be attributed to the beneficial activities of earthworms. Earthworms contribute to reducing pathogens by increasing enzyme activity in the digestion and elimination processes within their stomachs^39,40^. This reduction is closely linked to the activity of intestinal enzymes and the secretion of celomic enzymes with antibacterial properties. Alternatively, it may occur indirectly by stimulating microbial biomass and pathogens through earthworm movements. Notably, reducing pathogens during vermicomposting tends to be selective because different earthworm species possess varying capacities to inactivate pathogens^40,41^.

**Figure 6:**
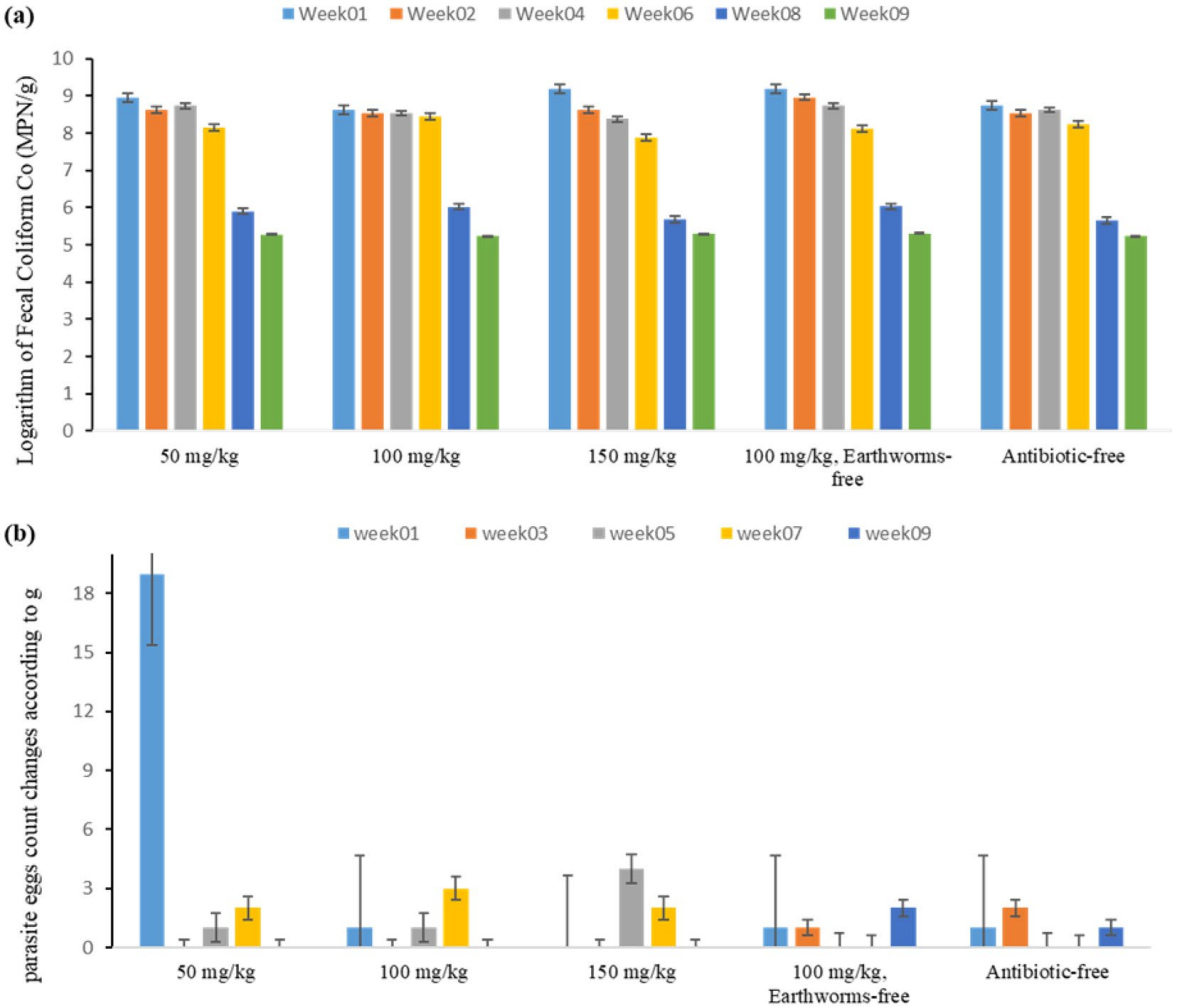
Observed variations in (**a**) fecal coliforms and (**b**) parasite egg throughout the vermicomposting duration in the studied reactors (50, 100, 150 mg/kg tylosin, 100 mg/kg tylosin without earthworm, and antibiotic-free reactor).

Moreover, the microbes left behind by earthworms in the compost have soil-enriching benefits. They compete with pathogenic organisms for limited nutrients, thus contributing to a healthier microbial community in the soil. Studies by Panikkar et al.^42^ revealed that earthworms secrete specific fluids with antibacterial properties. Dominguez and Edwards^43^ reported that human pathogens are not resistant to antibacterial fluids produced in vermicompost, and Panikkar et al.^42^ demonstrated the elimination of various human pathogens, including *Escherichia coli*, through composting^44^. According to the statistical analysis results, the type of reactor did not significantly affect the fecal coliform count (p = 0.213). In contrast, the duration of vermicomposting had a significant impact on changes in fecal coliform count (p < 0.001). Figure 6 illustrates the changes in the fecal coliform count in the studied reactors. As can be seen, a decreasing trend occurred from the first to the ninth week. In the survey by Karimi et al., a substantial decrease in the levels of fecal coliforms in cow manure-organic waste was observed^45^. Similarly, in the study by Aira et al., significant reductions in fecal coliforms and E. coli were reported, indicating that the reduction of pathogens depends on the specific type of pathogen^46^.

In this study, the evolution of parasite egg count over 8 weeks was investigated. As depicted in Fig. 6, initially, the count ranged from 0 to 19 eggs, reducing to between 0 and 2 eggs by the study's conclusion. Statistical analysis revealed that neither reactor type (P = 0.548) nor time (P = 0.529) significantly influenced the average parasite egg count. The standard for parasite eggs is less than 1.4 g of dry weight, and in our study, the count decreased to meet this standard by the study's end. Parseh et al. observed a complete elimination of parasite eggs after three weeks, attributing this to the varied effects of earthworm gut treatments on microbial populations. Factors such as the activity of earthworm intestinal enzymes, secretion of antibacterial cell fluid, and competition among microbial groups influence pathogen reduction during vermicomposting^40^. Eastman et al. demonstrated a significant decrease in parasite eggs through vermicomposting^47^, while Contreras-Ramos et al. reported the complete removal of parasite eggs and fecal coliforms via the same process^48^. Monory et al. also obtained results consistent with our findings^49^.

### Biodegradation of tylosin during vermicomposting

**Figure 7:**
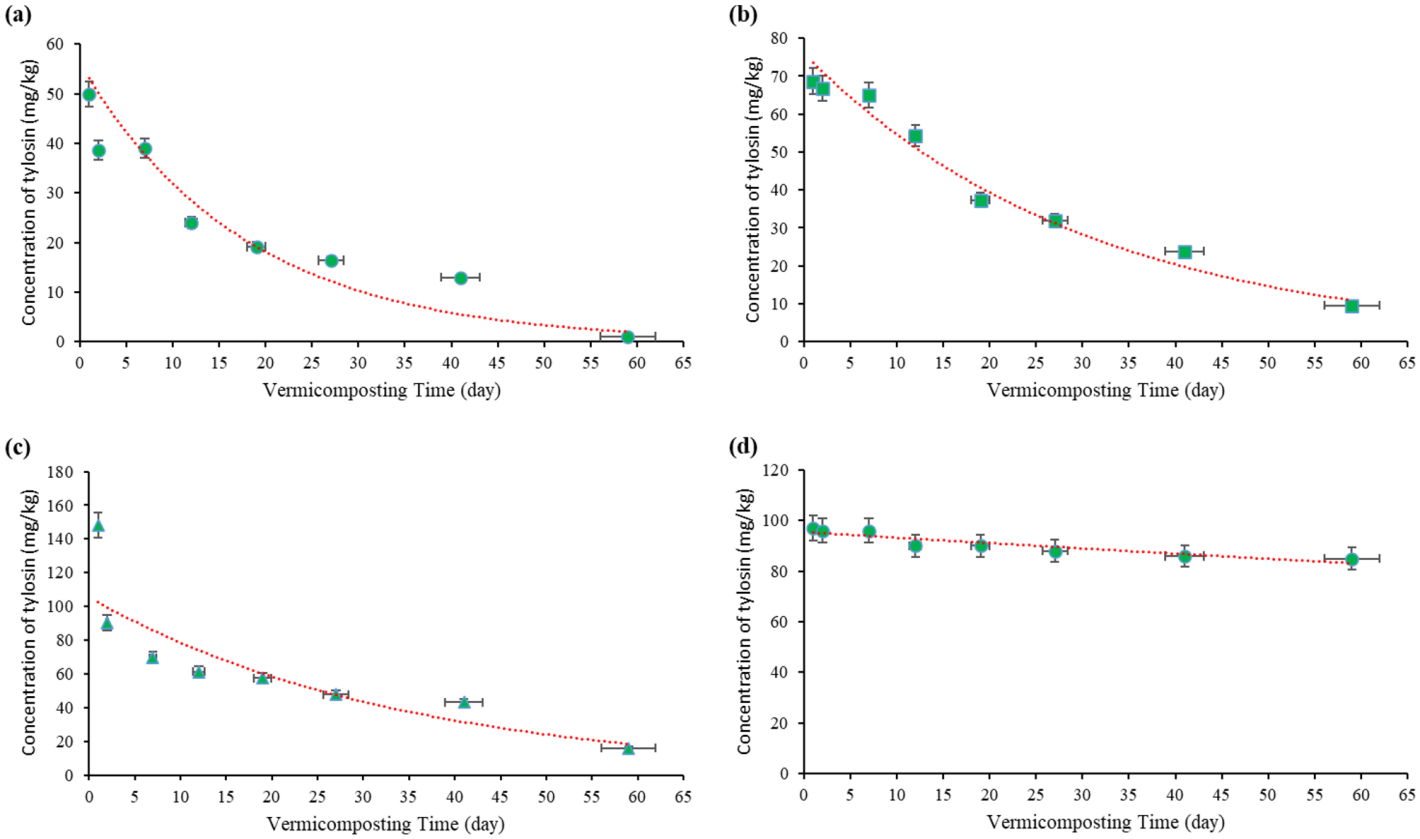
Biodegradation patterns of the different concentrations of tylosin by earthworm *Eisenia fetida* during the vermicomposting of cow manure: (**a**) 50 mg/kg tylosin, (**b**) 100 mg/kg tylosin, (**c**) 150 mg/kg tylosin, and (**d**) 100 mg/kg tylosin earthworm-free.

The changes in tylosin concentrations over the 9-week period of vermicomposting are presented in Fig. 7. It is evident that a decreasing trend was observed in reactors with concentrations of 50, 100, and 150 mg/kg tylosin. While the reactor type did not have a significant effect on the average tylosin concentration (p = 0.13), vermicomposting duration exhibited a substantial impact on tylosin removal efficiency (p < 0.001), with an observed increasing trend from the first to ninth week (Table 1). The removal efficiency in the reactors with concentrations of 50, 100, and 150 mg/kg tylosin at the end of the process (ninth week) was 98, 90.48, and 89.38%, respectively. This can depend on different parameters, including temperature, moisture content, substrate composition, and the initial antibiotic concentration^50^. During vermicomposting, earthworms lead to the effective removal of antibiotics by providing moisture, suitable temperature, and organic resources for microorganisms. Enzymes produced by earthworms may also play a role in breaking down chemical compounds that are challenging for microorganisms alone^51^. In previous studies, the effectiveness of composting and vermicomposting in removing veterinary antibiotics has been confirmed. Molavi et al. focused on co-trimoxazole during vermicomposting and demonstrated a reduction in its concentration^17^. Huang et al.^52^ investigated the impact of biochar on the fate of antibiotics and resistance genes during vermicomposting, achieving complete elimination of tetracycline^53^. Another study by Yang et al.^54^ addressed the removal of tetracycline chlorine and antibiotic-resistant genes in soil using earthworms. Their findings suggested that earthworms significantly reduced the presence of the antibiotic chlortetracycline and its metabolites in the soil^54^. Investigating the removal of metronidazole through composting, Jonidi Jafari et al. observed high removal efficiencies, with 99.9, 96.73, and 93.48% for reactors with concentrations of 20, 50, and 100 mg/kg, respectively. The removal efficiency increased with time, reaching 99.99% by the end of the process^9^. Chai et al. investigated the removal rate of tetracycline in pig manure during composting, revealing significant removal percentages for oxytetracycline, tetracycline, and chlorine tetracycline over 49 days^55^. Honglei et al.^56^ explored the change in antibiotic concentration during aerobic composting, noting over 90% removal at 55 °C^57^. Additionally, Lian Yang et al. demonstrated 100% destruction of penicillin antibiotic during composting^58^, and Zhang et al. found that aerobic co-composting effectively removed over 99% of penicillin after 7 days^58^. Youngquist et al. observed an 80% degradation of the antibiotic ciprofloxacin in the composting process after 28 days^59^. The presented findings align with the outcomes of these studies, emphasizing the potential of vermicomposting to reduce antibiotic residues in organic waste.

**Table 1:** Tylosin degradation efficiency by earthworm *Eisenia fetida* during vermicomposting.

Tylosin concentration (mg/kg)	Degradation constant (K)	R^2^	Removal efficiency (%)					
			First week	Second week	Third week	Fourth week	Sixth week	Ninth week
50	− 0.057	0.93	22.1	51.94	61.7	67.24	74.04	98
100	− 0.033	0.97	34.93	45.66	62.66	68.05	76.18	90.48
150	− 0.030	0.75	53.66	59.21	61.69	68.18	71.26	89.38

In this study, investigating the degradation constant across varying concentrations of tylosin indicates a dependence on first-order kinetics. The degradation constant for various tylosin concentrations ranged from 0.03 to 0.057 (Table 1). This can confirm the faster removal of tylosin in reactors with lower concentrations of antibiotics.

### The effect of tylosin concentration on the weight of earthworms

In this study, 120 g of *Eisenia fetida* earthworms were added to each reactor, and their initial weights were measured. After a 60-day period, the earthworms in each reactor were weighed again, and the results are presented in Table 2. According to statistical analysis, tylosin concentration had a significant effect on the weight of earthworms (p < 0.001). The weight of earthworms increased at the lowest antibiotic concentration and in the antibiotic-free control reactor. Conversely, a decreasing trend in earthworm weight was observed with an increase in antibiotic concentration. The reactor containing the lowest antibiotic concentration exhibited the highest weight gain. The growth, reproduction, and weight gain of earthworms serve as indicators of a successful vermicomposting process. These indicators are influenced by the organic substrates provided as a food source for earthworms. Therefore, the changes in worm biomass, number of worms, and cocoon production in the treatments indicate that the feed mixture impacts the growth and activity of earthworms^60^. In a study conducted by Boruah et al. an increase in the population of earthworms was observed, indicating a positive effect of mixing paper mill sludge with citronella bagasse on vermicompost production. The high speed of vermicompost production can be attributed to the active reproductive and metabolic activities of earthworms during pregnancy^23^. In Wang et al. study, the population of E. Eugenia worms increased gradually until the end of the vermicomposting process. The growth rate suggests that earthworm growth depends on the C/N ratio of the raw materials in the substrate^34^. Dominguez and Edwards observed a significant reduction in the organic nitrogen content and a high rate of nitrification by E. Andrei earthworms after two months of testing. After 8 months, the worms were larger and more active, indicating that earthworms, in this case E. Andrei, altered the conditions in the manure to aid nitrification, causing rapid ammonium conversion. Similar results were reported by Hand et al. using horse manure with *Eisenia fetida*^44^. In the study by Noor Mohammad Rasaei, the increase in the weight of earthworms after treating cow dung with palm leaves was attributed to the reproduction of more earthworms compared with other treatments. The study showed that the type of nutrition affects the growth of earthworms and their reproduction^61^.

**Table 2:** Observed variations in the weight of earthworms *Eisenia fetida* at different concentrations of tylosin.

Reactor containing tylosin (mg/kg)	Initial weight (g)	Final weight (g)	Changes (%)
50	120	135.61	+ 15.61
100	120	127.8	+ 7.8
150	120	115.65	− 4.35
Tylosin-free	120	135.96	+ 15.96

### Toxicity and maturity assessment

**Figure 8:**
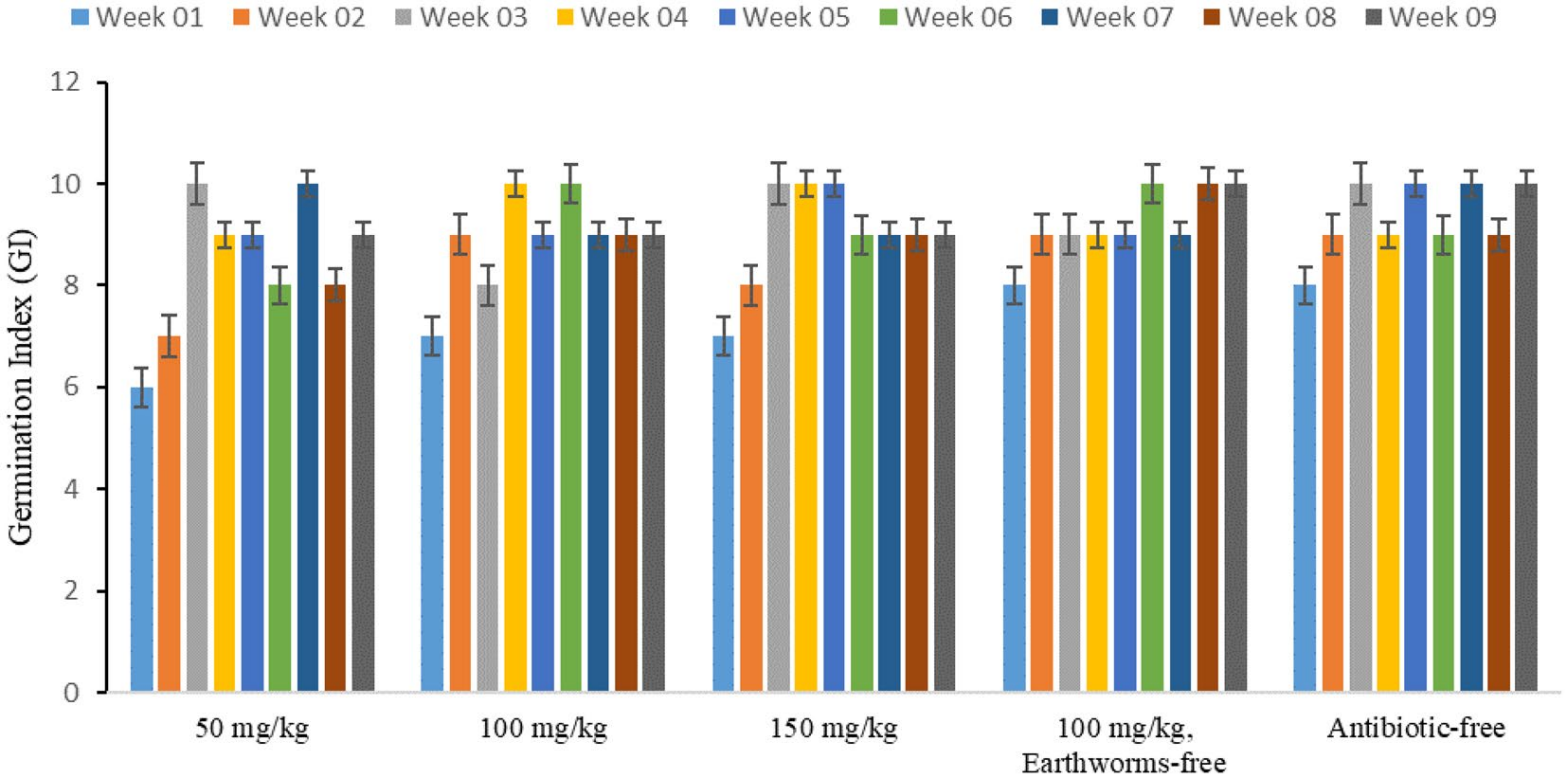
Observed variations in germination index throughout the vermicomposting duration in the studied reactors (50, 100, 150 mg/kg tylosin, 100 mg/kg tylosin without earthworm, and antibiotic-free reactor).

The germination index is a crucial and sensitive biological parameter for assessing the toxicity and maturity of vermicompost. It provides a comprehensive measure of both seed germination and root growth. A germination index value exceeding 80% indicates compost products that are free from phytotoxicity and fully mature. At the beginning of the study, except for the control reactor, the germination index ranged from 60 to 80% (Fig. 8). At the end of the process, the germination rate reached 90–100%. Comparing this to distilled water as a reference, follow-up tests indicated a significant difference in germination rates between reactors containing 150 mg/kg (p < 0.001), 100 mg/kg (p < 0.001), and 50 mg/kg (p < 0.001) of tylosin and the distilled water sample. Furthermore, in a second comparison with the reactor containing 100 mg/kg of tylosin but without earthworms as the reference, follow-up tests revealed a significant difference in germination rate between the reactor with 50 mg/kg of tylosin and the reference reactor (p = 0.019). In a third comparison, using the control reactor without antibiotics as the reference, follow-up tests showed a significant difference in germination rate between the reactor with 50 mg/kg of tylosin and the reference reactor (p = 0.008). In a study by Xiuren et al. a germination index of 86.42% was achieved, aligning with the results obtained in our study^13^. This consistency underscores the reliability of the germination index as an indicator of the quality and maturity of vermicompost.

## Conclusion

This study demonstrated the effectiveness of vermicomposting as a biological and cost-effective method for removing tylosin antibiotics. After 60 days of the vermicomposting, the removal efficiencies in the reactors with tylosin concentrations of 50, 100, and 150 mg/kg were 98.90.48 and 89.38%, respectively. The presence of earthworms in the vermicomposting was effective in antibiotic removal. The physicochemical analysis at the end of the process indicated that various parameters, including Kjeldahl nitrogen, electrical conductivity, acidity, organic carbon, C/N, volatile solids, toxicity, and fecal coliforms, met the national standards of Iran. Moreover, the resulting fertilizer from the vermicomposting adhered to the quality standards for compost in Iran, confirming the environmental friendliness of this method. In conclusion, vermicomposting can be considered an effective and environmentally friendly method for removing antibiotics from organic waste, providing a valuable and compliant fertilizer as the end product.

## Data Availability

The supporting data are available from the corresponding authors upon reasonable request.
